# The development of endometrial hyperplasia in aged PD-1-deficient female mice

**DOI:** 10.1186/1746-1596-9-97

**Published:** 2014-05-26

**Authors:** Guoning Guo, Hong Li, Dayan Cao, Yongwen Chen

**Affiliations:** 1 Department of Emergency, South-West Hospital, Third Military Medical University, Chongqing 400038, China; 2 Department of Otorhinolaryngology and Head-Neck Surgery, Xinqiao Hospital, PLA, Third Military Medical University, Chongqing 400037, PR China; 3 Institute of Immunology, PLA, Third Military Medical University, Chongqing 400038, People’s Republic of China

**Keywords:** PD-1, Endometrial hyperplasia, VEGF, PCNA, Uterus

## Abstract

**Background:**

Programmed death-1 (PD-1, *Pdcd1*)-deficient mice develop different types of autoimmune diseases depending on the mouse strain but its role in uterus development has not been reported.

**Methods:**

In this study, the expression of PD-1 and its ligands, PD-L1 and PD-L2, in uterine tissues from aged WT mice in a 129svEv-Brd background was analyzed by immunohistochemistry and the uterine morphology between WT and PD-1^-/-^ mice was compared by hematoxylin and eosin staining.

**Results:**

The aged PD-1^-/-^ female mice in a 129svEv-Brd rather than Balb/c background develop endometrial hyperplasia. H&E staining showed an increase in the number of glands, neovascularization and an extremely large luminal cavity in aged PD-1^-/-^ uteri. Immunohistochemical assay showed that the expression of PD-1 was observed in glandular/luminal epithelium and cells infiltrating the stroma. Fluorescent double staining demonstrated that PD-1 expresses on CD68^+^ macrophages, CD3^+^ T cells, CD16^+^ monocytes, CD56^+^ NK cells and CK-18^+^ epithelial cells, respectively. Additionally, PD-1 co-expresses with vascular endothelial growth factor (VEGF), and PD-1 deficiency resulted in an accumulation of glandular/luminal epithelium derived VEGF, which accelerates the expression of the proliferation-associated protein, proliferating cell nuclear antigen (PCNA), and thus potentially lead to epithelial proliferation in aged PD-1^-/-^ uteri.

**Conclusions:**

These findings showed that PD-1 deficiency augments luminal epithelial cell proliferation probably through induced VEGF secretion, suggesting PD-1 plays an important role in controlling the growth and differentiation of the uterine epithelium.

**Virtual Slides:**

The virtual slide(s) for this article can be found here: http://www.diagnosticpathology.diagnomx.eu/vs/5809067461223905

## Background

Co-signaling by B7/CD28 family members regulates the initiation, maintenance, and termination of immune responses. Programmed death-1 (PD-1) is an inhibitory receptor expressed on activated T cells, B cells and myeloid cells [1]. PD-1 deficiency (PD-1^-/-^) causes lupus-like glomerulonephritis and arthritis in C57BL/6 mice [2,3], autoimmune dilated cardiomyopathy (DCM) and gastritis in BALB/c mice [4,5], acute type 1 diabetes mellitus (T1DM) in nonobese diabetic (NOD) mice [6], and lethal myocarditis in MRL mice [7]. In humans, polymorphisms in the PD-1 gene have been associated with susceptibility to systemic lupus erythematosus [8], type I diabetes [9], multiple sclerosis [10], and rheumatoid arthritis [11]. Additionally, PD-1^-/-^ mice in a 129svEv-Brd background were also more susceptible to the development of experimental autoimmune encephalomyelitis (EAE) [12]. Nevertheless, the organ development regulated by PD-1 signal is still under investigation.

PD-L1 (B7-H1) and PD-L2 (B7-DC), two immunoregulatory molecules belonging to the B7 family, were identified as the ligands for PD-1 [13,14]. The expression of PD-L1 has been detected not only in lymphoid organs but also in nonlymphoid tissues and was enhanced in several types of tumor cells under inflammation conditions, suggesting that PD-L1 might regulate lymphocyte function at sites of inflammation [15]. The expression of PD-L2, however, was restricted in activated dendritic cells (DCs), macrophages, monocytes and T cells [16].

The expression, anatomic distribution and potential role for PD-1/PD-Ls in uterine development have not been investigated. We here showed that aged PD-1-deficient female mice in a 129svEv-Brd background develop endometrial hyperplasia. This effect potentially reflects the induction VEGF secretion from epithelial cells upon PD-1 signaling deficiency.

## Methods

### Ethics statement

All experiments were approved and conducted in accordance with the guidelines of the Animal Care and Use Committee of the Third Military Medical University. All efforts were made to minimize animal suffering.

### Mice

PD-1-deficient mice (Background: 129svEv-Brd) were kindly provided by Dr. Laura L. Carter (Inflammation Department, Wyeth Research, Cambridge, MA, USA). Prof. T. Honjo (Department of Immunology and Genomic Medicine, Kyoto University, Japan) kindly gave us the PD-1-KO-N10 mice (strain: BALB/cJ). The WT control mice were purchased from the Animal Center of Beijing University School of Medicine. All mice were maintained in micro-isolator cages and housed in the animal colony at the Animal Center, Third Military Medical University, and standard laboratory chow diet and water was supplied.

### Histology and immunohistochemistry

Section were used to detect the indicted protein expression with using the following primary antibodies: anti-PD-L1 (2.5 μg/ml, Catalog#: AF1019, R&D Systems), anti-PD-L2 (2.5 μg/ml, Catalog#: AF1022, R&D Systems), anti-PD-1 (2 μg/ml, Catalog#: AF1021, R&D Systems) and anti-VEGF (1:100, clone: C-1, Santa Cruz). A previously published protocol for immunohistochemistry was used [17].

### Immunofluorescent double staining

For immunofluorescent double staining, the sections were incubated with mouse monoclonal anti-PD-1 and anti-VEGF antibodies at 4°C overnight. After washing with PBS (3x5-min incubations), sections were incubated with Alexa 568-conjugated goat anti-mouse IgG antibodies (Jackson ImmunoResearch, West Grove, PA, USA) for 1 h. Sections were subsequently further incubated with anti-CD3 (1:50, Abcam), anti-CD56 (1: 150, Santa Cruz), anti-CK-18 (1: 150, clone: H-80, Santa Cruz), anti-CD68 (1: 200, clone: H-255, Santa Cruz) antibodies at 4°C overnight and incubated with fluorescent isothiocyanate-conjugated goat anti-mouse IgG antibodies (Jackson ImmunoResearch) for an additional 1 h. Subsequently, the sections were incubated with 1 μg/ml 4′,6-diamidino-2-phenylindole (DAPI, Sigma, CA, USA) for 10 min to stain the nuclei. Sections incubated with the appropriate isotype control primary antibodies and fluorescently labeled secondary antibodies were used as negative controls.

### Cell count and statistical analysis

The proportion of PCNA-positive nuclei in the glandular epithelium was determined through image analysis of the histological sections. Photomicrographs were captured and analyzed using Image Pro-Plus 5.0 software (Media Cybernetics, Silver Spring, MD) [18]. The number of PCNA^+^ nuclei per high-power field was counted. The data were analyzed using GraphPad Prism 4.03 software. An unpaired Student *t* test (two-tailed) was used to assess comparisons of PCNA^+^ nuclei between PD-1^-/-^ and WT uteri. A *p* value <0.05 was considered statistically significant different.

## Results

### Changes in the gross anatomy and morphology of the uteri in aged PD-1^-/-^ female mice

**Figure 1:**
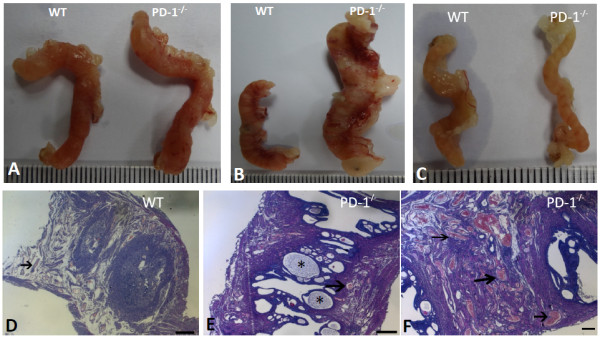
**Gross anatomic and histological comparison of uteri between PD-1**^**-/- **^**and WT mice.** Gross anatomy of uteri between PD-1^-/-^ and WT mice (129svEv-Brd background) at **(A)** 1 year and **(B)** 2 years of age, results showed that uteri from PD-1^-/-^ mice in 2-year old are more larger than WT littermates. One of represents five mice was shown. **(C)** Gross anatomy of uteri from PD-1^-/-^ and WT mice on Balb/c background at 2-year age was compared, results showed that uteri from PD-1^-/-^ mice were similar to than from WT littermates. One of represents five mice was shown. Histology of uteri from WT **(D)** and PD-1^-/-^ mice **(E and F)** in a 129svEv-Brd background at 2-year age was compared by using H&E staining. Star indicated glands and arrow showed neovascularization; **D-F**, Scale bar = 20 μm; N = 5 of each group.

The WT and PD-1^-/-^ mice at different ages were sacrificed, and the morphology of several organs was compared. An interesting finding is the uteri from PD-1^-/-^ female mice in the 129svEv-Brd rather Balb/c background are much larger than that of the WT littermates at 2, 2.5 and 3 years of age (Figure 1B and C), indicating that the aged PD-1^-/-^ female mice developed endometrial hyperplasia. Nevertheless, the uteri from young (for example, 1-year-old) PD-1^-/-^ and WT mice in 129svEv-Brd background were comparable (Figure 1A). H&E staining showed that there were numerous glands (Figure 1E) and neovascularizations (Figure 1F) in the aged PD-1^-/-^ uteri (129svEv-Brd background), as compared to WT controls at 2 years of age (Figure 1D). These findings suggested that endometrial hyperplasia was developed in aged PD-1^-/-^ female mice in a 129svEv-Brd background.

### PD-1 deficiency resulted in epithelial cell proliferation

**Figure 2:**
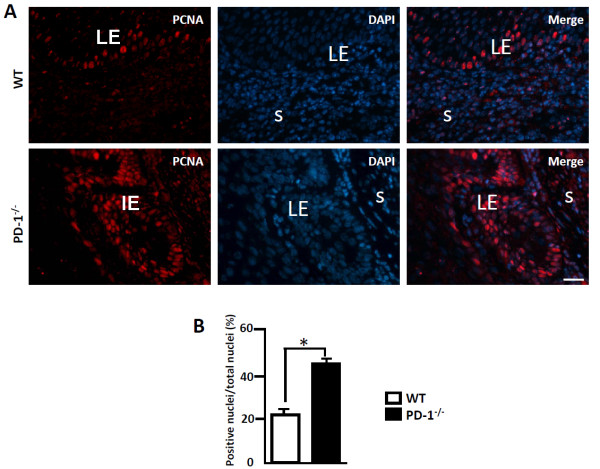
**Enhanced PCNA positive cells in PD-1**^**-/- **^**uteri was measured by immunofluorescent staining.** Uteri from 2-year old PD-1^-/-^ and WT mice were selected to detect the expression of PCNA by immunofluorescent staining **(A)**. LE: luminal epithelium; S: stroma. The blue color indicates nuclear staining with 4′,6-diamidino-2-phenylindole (DAPI). Scale bar = 20 μm. N = 4 of each group. **(B)** The PCNA-positive nuclei in uterine tissues between PD-1^-/-^ and WT mice were counted and compared. *p < 0.05.

To analyze the pathogenesis of endometrial hyperplasia in aged PD-1^-/-^ female mice, the expression of the proliferation-associated protein, proliferating cell nuclear antigen (PCNA), was analyzed in aged uteri between PD-1^-/-^ and WT mice in 129svEv-Brd background. Immunofluorescent staining revealed the presence of PCNA^+^ cells in the uteri, and PCNA expression was primarily detected in luminal/glandular epithelium (Figure 2A). The PCNA^+^ nuclei were counted and results showed that 42.1 ± 3.2% of luminal/glandular epithelial cells in PD-1^-/-^ uteri were positive for PCNA, while only 21.4 ± 1.2% of the cells in WT uteri showed nuclei positive for PCNA (Figure 2B), suggesting that PD-1 deficiency triggers epithelial cell proliferation in the aged uteri.

### Detection and localization of PD-1 and its ligands in the aged uteri

**Figure 3:**
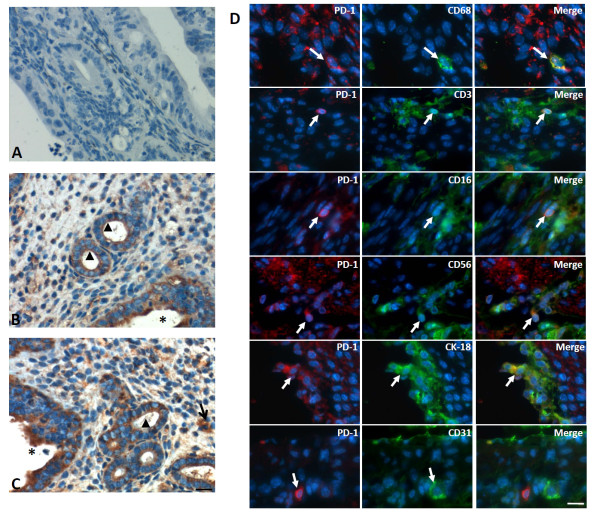
**The characteristic and anatomic distribution of PD-1 protein in uteri from WT mice was detected by immunohistochemistry and immunofluorescent double staining.** Uteri from 2-year old PD-1^-/-^ and WT mice were selected to detect the expression of PD-1 by Immunohistochemistry. **(A)** Uteri from 2-year old PD-1^-/-^ mice showed no positive staining; However, uteri from 2-year old WT mice showed PD-1-positive cells were observed in glandular/luminal epithelium **(B)** and filtrating the stroma **(C)**, ▲indicates the glandular epithelium; *indicates the luminal cavity and the arrows indicate positive cells infiltrating the stroma. **(D)** immunofluorescent double staining showed that PD-1 was expressed on CD68^+^ macrophages, CD3^+^ T cells, CD16^+^ monocytes, CD56^+^ NK cells and CK-18^+^ epithelial cells, but PD-1 expression was absence on CD31^+^ endothelial cells. The blue color indicates nuclear staining with 4′,6-diamidino-2-phenylindole (DAPI), arrows indicate positive cells. Scale bar = 20 μm. N = 4 of each group.

We further analyzed the expression of PD-1 and its ligands, PD-L1 and PD-L2 in uteri of aged WT mice (129svEv-Brd background). Immunohistochemistry revealed that PD-1 protein was expressed in glandular and luminal epithelium (Figure 3B). However, uterine sections from aged PD-1^-/-^ mice incubated with anti-mouse PD-1 antibodies were absence PD-1 expression (Figure 3A). Some cells infiltrated in stroma were also positive for PD-1 (Figure 3C). Immunofluorescent labeling showed PD-1 was presence on CD68^+^ macrophages, CD3^+^ T cells, CD16^+^ monocytes, CD56^+^ NK cells and CK-18^+^ epithelial cells, but it was absent on CD31^+^ endothelial cells (Figure 3D).

**Figure 4:**
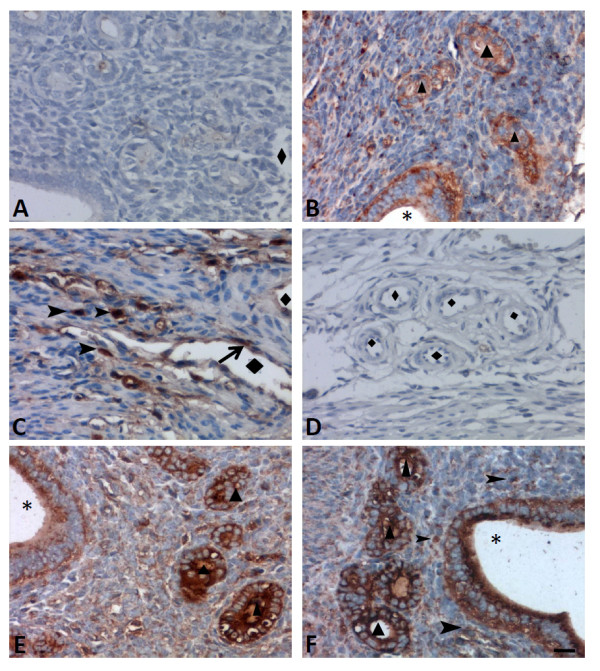
**The characteristic and anatomic distribution of PD-1 ligands, PD-L1 and PD-L2, in uteri from 2-year old WT mice was detected by immunohistochemistry. (A)** Mouse IgG1 isotype control antibodies showed no positive staining; **(B)** PD-L1 positive cells were observed in the glandular/luminal epithelium; **(C)** PD-L1 positive cells were observed in the endothelium and cells infiltrated stroma; **(D)** The PD-L2 expression was absent in blood capillary; **(E)** The PD-L2 positive cells were observed in the glandular/luminal epithelium; and **(F)** PD-L2 positive cells were observed infiltrating the stroma. ▲ indicates the glandular epithelium; *indicates the luminal cavity; ◆ indicated blood capillary. The arrows indicate positive endothelial cells and arrow head showed infiltrated cells that are positive for PD-L1 or PD-L2. Scale bar = 20 μm. N = 4 of each group.

The expression of the PD-1ligands, PD-L1 and PD-L2, in the aged uteri from WT mice was also detected by immunohistochemistry. Similar to PD-1, the expression of PD-L1 was also observed in glandular/luminal epithelium (Figure 4B). Cells infiltrating the stroma and some endothelial cells were also positive for PD-L1 (Figure 4C). In contrast, the expression of PD-L2 was absent in blood endothelium (Figure 4D) but present in glandular/luminal endothelium and cells infiltrating the stroma (Figure 4E and F).

### Augmented VEGF secretion in uteri from aged PD-1 deficient mice

**Figure 5:**
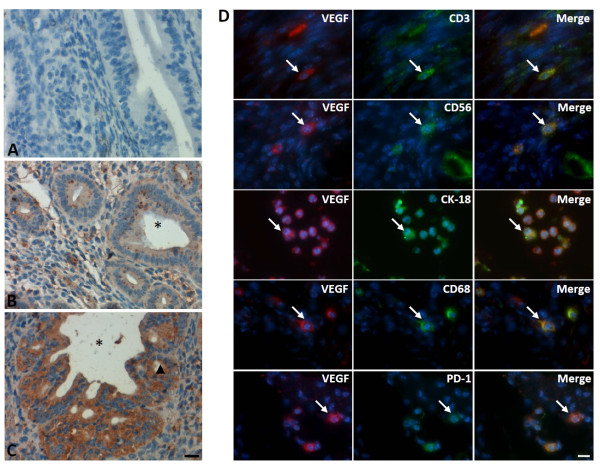
**The characteristic and anatomic distribution of VEGF in uteri from 2-year old WT and PD-1**^**-/- **^**mice.** Immunohistochemistry detection showed **(A)** no mouse positive staining using IgG isotype control antibodies; **(B)** the VEGF-positive cells were weakly observed in the glandular/luminal epithelium of WT uteri; **(C)** Strong VEGF-positive staining was observed in the glandular/luminal epithelium from PD-1^-/-^ uteri. ▲ indicates the glandular epithelium; *indicates the luminal cavity and arrows showed the positive cells; **(D)** immunofluorescent double staining showed that VEGF is expressed on CD3^+^ T cells, CD56^+^ NK cells, CK-18^+^ epithelial cells, CD68^+^ macrophages and PD-1^+^ cells. The blue color indicates nuclear staining with 4′,6-diamidino-2-phenylindole (DAPI), and arrows showed the positive cells. Scale bar = 20 μm. N = 4 of each group.

To analyze the potential molecular mechanism for epithelial cell proliferation in aged PD-1^-/-^ uteri, the expression of VEGF, a growth factor essential for cell proliferation and uterine growth, was compared between uteri from PD-1^-/-^ and WT mice in 2 years old. Immunohistochemistry showed that the expression of VEGF was slightly detected in uteri from WT mice (Figure 5B). However, strong VEGF expression was observed in uteri from aged PD-1^-/-^ female mice, and the expression was primarily observed in the luminal epithelium and cells infiltrated in stroma (Figure 5C). Immunofluorescent labeling showed that VEGF was co-expressed with PD-1^+^ cells, in addition to it expresses on CD68^+^ macrophages, CD3^+^ T cells, CD16^+^ monocytes, CD56^+^ NK cells and CK-18^+^ epithelial cells (Figure 5D). These results suggest that PD-1 deficiency likely stimulates luminal epithelial cell proliferation through induced VEGF secretion.

## Discussion

The disruption of the PD-1 signal leads to the breakdown of peripheral tolerance and the initiation of autoimmunity like dilated cardiomyopathy. This effect is due to PD-1 negatively controls T cell receptor (TCR) signaling [19]. Recently, it was shown that PD-1 deficiency accelerates microRNA-21 (miR-21) overexpression, thus lead to cell proliferation through the enhanced expression of programmed cell death 4 (PDCD4) [20]. Here, we provided the first evidence that endometrial hyperplasia was developed in aged PD-1^-/-^ female mice in a 129svEv-Brd rather than Balb/c background (Figure 1B), suggesting PD-1 plays an important role in the growth and differentiation of the uterine epithelium in129svEv-Brd mice. Our results further reflect that the development of disease mediated by PD-1 signals in animal models is strain restricted.

The expression of PD-1 has been reported on T cells, natural killer T cells, B cells and monocytes, and this expression was enhanced through stimulation with inflammatory factors, such as TNF-α and IFN-γ. However, PD-L1 and PD-L2, the two PD-1ligands, shows different expression patterns [21]. Here, we showed that the expression of PD-1 was observed on glandular and luminal epithelium (Figure 3B) and cells infiltrated in stroma (Figure 3C). Additionally, PD-1 was also detected to be expressed on CD68^+^ macrophages, CD3^+^ T cells, CD16^+^ monocytes, CD56^+^ NK cells and CK-18^+^ epithelial cells, but it was absent on CD31^+^ endothelial cells, as detected by immunofluorescent double staining (Figure 3D). Additionally, the expression of PD-L1 and PD-L2 was also detected on luminal epithelium and infiltrating cells within the aged uterine tissues (Figure 4). Due to strong neovascularization (Figure 1F) and higher level of PCNA^+^ cells were seen in aged PD-1^-/-^ uteri, suggesting strong cell proliferation is in progress in PD-1^-/-^ uteri (Figure 2). These combine data suggest that local PD-1/PD-Ls signal probably controls glandular/luminal epithelial biofunction, like cell proliferation and neovascularization. Indeed, the expression of PD-1 on the tubular epithelium of murine Adriamycin nephropathy (AN) has been reported previously, and blockade of PD-1 worsened progressive renal histopathological and functional injury in murine AN [22]. Taken together, our results suggested that PD-1 is not only expressed on immune cells but also on nonlymphoid tissues, and uterine local PD-1/PD-Ls signal probably directly inhibits glandular/luminal epithelial cell proliferation and neovascularization.

To analyze other potential molecular mechanisms that involve in the growth and differentiation of the uterine epithelium in aged PD-1^-/-^ mice, the secretion of VEGF, which stimulate endothelial and epithelial cell proliferation through its receptor, VEGFR, was compared in aged uteri between PD-1^-/-^ and WT mice. An interesting finding is that glandular/luminal epithelium derived-VEGF in aged uteri from PD-1^-/-^ mice was augmented dramatically (Figure 5C), thereby potentially promotes cell proliferation *via* its receptor VEGFR and thus resulted in accelerating neovascularization (Figure 1F). Additionally, VEGF in uteri was also co-expresses with PD-1 (Figure 5D), suggesting that PD-1 signaling inhibits epithelial cell proliferation potentially through a reduction of VEGF secretion, in addition to direct prevents epithelial proliferation by cross-reacts with PD-Ls. However, the expression of PTEN, a tumor suppress gene, were significantly higher in cyclical endometrium than in atypical hyperplasia and endometrioid carcinoma, indicated that PTEN involves in the pathogenesis of endometrial hyperplasia [23]. On the other hand, CyclinD1, a cell -cycle regulator, exhibited a promising potential to predict the prognosis of patients with endometrial carcinoma [24]. Whether the transcription of PTEN or CyclinD1 is also controlled by PD-1/PD-Ls need further investigation.

## Conclusion

PD-1 deficiency augments luminal epithelial cell proliferation and neovascularization in aged uteri, suggesting that PD-1 plays an important role in the organization, growth and differentiation of the uterine epithelium.

## Competing interests

The authors declare that they have no competing interests.

## Authors’ contributions

GG is responsible for immunohistochemistry, HL participated in the discussion for histological diagnosis and manuscript preparation. DC was responsible for immunofluoresent double staininge and YC is preparing the manuscript. All authors read and approved the final manuscript.
